# A Sex‐Specific Trade‐Off Between Pesticide Resistance and Tolerance to Heat‐Induced Sterility in *Tetranychus urticae*

**DOI:** 10.1111/eva.70014

**Published:** 2024-09-26

**Authors:** Sofia G. Costa, Sara Magalhães, Inês Santos, Flore Zélé, Leonor R. Rodrigues

**Affiliations:** ^1^ Centre for Ecology, Evolution and Environmental Changes & CHANGE – Global Change and Sustainability Institute (cE3c), Faculty of Sciences University of Lisbon Lisbon Portugal; ^2^ Institute of Evolution Sciences (ISEM), CNRS, IRD, EPHE University of Montpellier Montpellier France

**Keywords:** climate warming, crop pests, fertility, reproductive success

## Abstract

Current pest management relies extensively on pesticide application worldwide, despite the frequent rise of pesticide resistance in crop pests. This is particularly worrisome because resistance is often not costly enough to be lost in populations after pesticide application, resulting in increased dependency on pesticide application. As climate warming increases, effort should be put into understanding how heat tolerance will affect the persistence of pesticide resistance in populations. To address this, we measured heat tolerance in two populations of the spider mite crop pest *Tetranychus urticae* that differ in the presence or absence of a target‐site mutation conferring resistance to etoxazole pesticide. We found that developmental time and fertility, but not survival, were negatively affected by increasing temperatures in the susceptible population. Furthermore, we found no difference between resistant and susceptible populations in all life‐history traits when both sexes developed at control temperature, nor when females developed at high temperature. Resistant heat‐stressed males, in contrast, showed lower fertility than susceptible ones, indicating a sex‐specific trade‐off between heat tolerance and pesticide resistance. This suggests that global warming could lead to reduced pesticide resistance in natural populations. However, resistant females, being as affected by high temperature as susceptible individuals, may buffer the toll in resistant male fertility, and the shorter developmental time at high temperatures may accelerate adaptation to temperature, the pesticide or the cost thereof. Ultimately, the complex dynamic between these two factors will determine whether resistant populations can persist under climate warming.

## 1 Introduction

Crop pests are responsible for enormous economic losses worldwide (Culliney 2014; Oliveira et al. 2014). Particularly, insects and mites account for most of the damage to crops, with estimated losses of 10%–20% preharvest and 5%–10% postharvest (Culliney 2014). Current pest management relies extensively on pesticide application, despite its unambiguous adverse effects (IPBES 2019; Janssen and van Rijn 2021; Jacquet et al. 2022). Indeed, the use of pesticides is one of the main drivers of biodiversity loss, including of biological control agents (Geiger et al. 2010; Sánchez‐Bayo and Wyckhuys 2019; IPBES 2019), and it is highly detrimental to the environment and human health (Bourguet and Guillemaud 2016; Kim, Kabir, and Jahan 2017). The evolution of pesticide resistance in crop pests (Bass et al. 2015; Hawkins et al. 2019) along with the loss of biocontrol agents and other ecosystem services, in turn, increases the dependency on pesticide application to ensure pest control, resulting in the emergence of a ‘pesticide treadmill’ (van den Bosch 1989).

Current climate warming is leading to a continuous increase in intensity and frequency of temperature extremes on a global scale (Seneviratne et al. 2021). The response of arthropod crop pests to high temperature can lead to increased or decreased crop losses depending on the pest species and other varying biotic and abiotic factors (Skendžić et al. 2021). For instance, several insects show reduced physiological performance at extreme temperatures (Zou et al. 2018; Lehmann et al. 2020; Rodrigues, McDermott, et al. 2022; Costa, Magalhães, and Rodrigues 2023), which may alleviate crops (Lehmann et al. 2020). In contrast, many crop pests respond to high temperatures by increasing in number (Lehmann et al. 2020) due, for example, to a rise in metabolic rate and population growth (Deutsch et al. 2018), or to the disruption of biological control as biocontrol agents show lower tolerance to high temperatures (Montserrat, Sahún, and Guzmán 2013). When this increase in pest number occurs, it may be necessary to increase pesticide use (Delcour, Spanoghe, and Uyttendaele 2015).

Given that pest management greatly depends on pesticide application, effort should be put into understanding the reciprocal interaction between heat tolerance and pesticide resistance, such as to determine the persistence of pesticide resistance under climate warming. In fact, even if agricultural practices are moving towards a reduction in the use of pesticides, resistant genotypes can remain in the environment for many generations (Kontsedalov et al. 1998; Bielza et al. 2008), especially those that are not costly in pesticide‐free environments (Bielza et al. 2008; Abbas et al. 2014). How these genotypes will respond to climate change is thus key to understand the effect of climate change on crop pest populations. Various studies have shown that resistance to pesticide can be reduced under heat stress (reviewed in Matzrafi 2019). However, evidence on whether heat tolerance differs between resistant and susceptible individuals in a pesticide‐free environment is rare (but see Fulton et al. 2021; Langmüller et al. 2020; Li et al. 2007; Ma et al. 2021; Yang et al. 2018; Zhang et al. 2015). This is unfortunate because such knowledge is crucial to predict the dynamics of crop pests under climate warming given that, depending on the outcome, one could expect the maintenance or loss of pesticide resistance in a population and thus the failure or success of pesticide applications, respectively.

Increase in the efficacy of pesticides under global warming would be expected when pesticide resistance and heat tolerance trade‐off, given the expected decline in the number of resistant individuals in such a scenario. This has been shown in a few studies (Li et al. 2007; Zhang et al. 2015; Yang et al. 2018). For example, in the diamondback moth *Plutella xylostella*, chlorpyrifos‐resistant individuals have significantly lower survival, adult emergence rates and higher damages of wing veins under heat stress than susceptible ones (Zhang et al. 2015). In turn, the efficacy of pesticides is expected to decrease whenever resistant individuals are also more tolerant to heat stress than susceptible ones, meaning they will spread quicker in the population under climate warming. For example, in *Drosophila simulans*, differences in viability between haplotypes susceptible and resistant to carbamate and organophosphate are reduced at high temperatures; thus, the resistant haplotypes have lost their disadvantage compared with susceptible ones under these conditions (Langmüller et al. 2020).

The majority of studies focusing on the costs of pesticide resistance disregard the possibility of sex‐specific responses. This is unfortunate, as the meagre evidence of such a phenomenon call attention to its important implications. For example, in *Drosophila melanogaster*, resistance to DDT is costly in males but increases female reproductive output in the absence of pesticide (Smith et al. 2011), which helps explaining why the resistance allele was present in populations at low frequency prior to the widespread use of DDT (Rostant et al. 2015). Likewise, whereas thermal tolerance studies mostly focus on survival estimates, extensive work has demonstrated that understanding the tolerance to temperature of fertility is also crucial to accurately predict the impact of climate change on organisms (Parratt et al. 2021; Walsh et al. 2019). Moreover, evidence of sex‐specific thermal sensitivity in different taxa is accumulating (Vasudeva, Deeming, and Eady 2014; Iossa 2019), with reproductive traits being affected at lower temperatures in males than in females (e.g., David et al. 2005; Iossa 2019; Rodrigues, Zwoinska, et al. 2022; Sales et al. 2018; van Heerwaarden and Sgrò 2021). As such, male, not female, thermal fertility limits have been identified as the best predictors of the response of populations to climate change (Parratt et al. 2021; van Heerwaarden and Sgrò 2021). In haplodiploid species, heat‐induced sterility can also lead to variation in sex ratio and productivity, depending on the sex that is being affected (Costa, Magalhães, and Rodrigues 2023). Indeed, because fertilised eggs only generate daughters, whereas sons develop from unfertilised eggs, male sterility should lead to a more male‐biased sex ratio and female sterility to fewer offspring. Such sex‐specific responses are bound to affect plastic and/or evolutionary responses of populations (Costa, Magalhães, and Rodrigues 2023). Therefore, disregarding sex‐specific responses compromises our ability to accurately predict the impact of climate warming on the persistence of pesticide resistance in populations. Yet, to date no study has investigated the existence of such sex‐specific responses.

Here, we tested the effect of high temperature and its interaction with pesticide resistance on three life‐history traits (survival, developmental time and fertility) in *Tetranychus urticae*. This spider mite is a haplodiploid polyphagous agricultural pest that occurs on a wide variety of plant species across the world, some of which of great economic significance (Migeon, Nouguier, and Dorkeld 2010). Pesticides are commonly used to control the spread of mite population in crops, but the success of this control strategy is often compromised by the spider mites' exceptional ability to rapidly evolve resistance (Van Leeuwen et al. 2010). We have created two populations with the same genetic background but a different resistance status (susceptible vs. resistant) to etoxazole, an acaricide commonly used on populations of this species worldwide (Kim et al. 2008). This allows us to attribute any observed differences between populations to the resistance allele or to changes in the genome due to its presence, rather than to other differences between populations. Etoxazole resistance has been thoroughly characterised in *T. urticae* (Van Leeuwen et al. 2009, 2012): this pesticide exhibits high efficacy as a chitin biosynthesis inhibitor in *T. urticae* during the developmental stages, being lethal to eggs and juveniles, but not adults (Nauen and Smagghe 2006; Van Leeuwen et al. 2012) and resistance is recessive and conferred by a single nonsynonymous mutation affecting the chitin synthase enzyme (Van Leeuwen et al. 2012). Despite this wealth of information, no study has yet tested if the resistance status of each sex elicits specific responses to temperature. Here, we set out to fill this gap.

## 2 Materials and Methods

### 2.1 Spider Mite Populations

**FIGURE 1:**
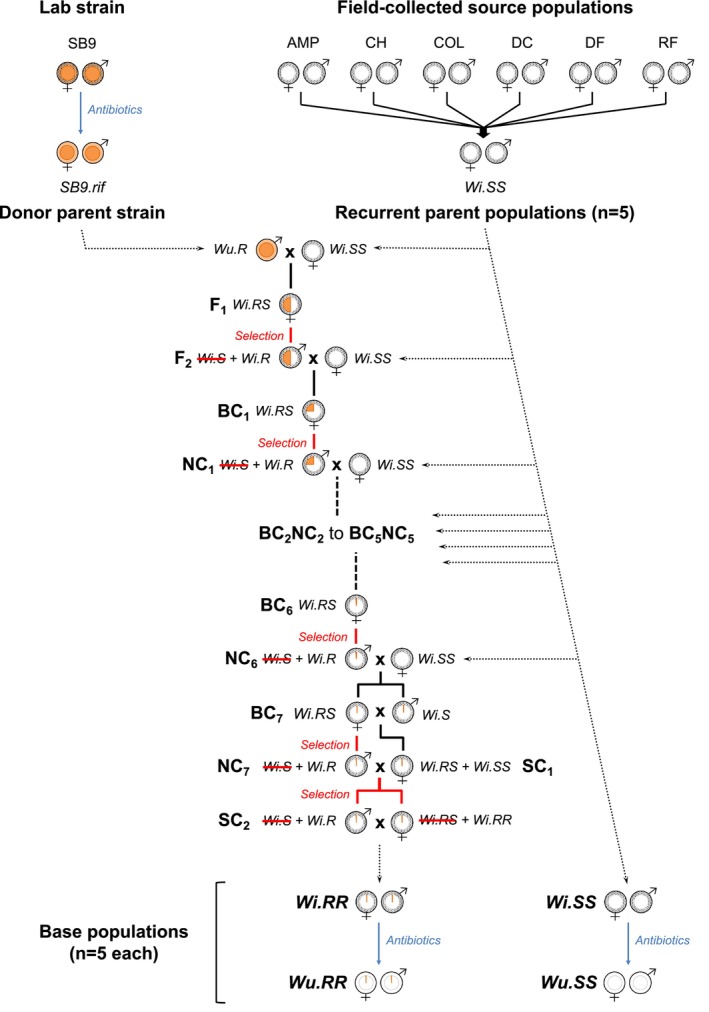
Backcross procedure used to introgress the etoxazole‐resistant allele into the susceptible *Wolbachia*‐infected populations *Wi.SS* to create the resistant populations *Wi.RR*, and subsequent antibiotic treatment to obtain the *Wolbachia*‐uninfected populations *Wu.RR* and *Wu.SS*. The entire procedure, from the creation of the *Wi.SS* populations to the obtention of all base populations, was performed in five independent replicates. *Wu.RR* and *Wu.SS* replicates were then merged before being used in the current study. Antibiotic treatments are represented in blue, and selection for etoxazole resistance is in red. Inner and outer circles within male/female symbols represent, respectively, the proportion of nuclear and mitochondrial genomes from the donor (orange) and recurrent (white) parent populations. Dotted‐filled outer circles represent *Wolbachia*‐infected cytoplasm. BC, backcross progeny; NC, no‐cross progeny; SC, Self‐cross progeny.

Two populations of the spider mite *T. urticae* red form (a.k.a. *T. cinnabarinus*; Cruz et al. 2021) that share the same genetic background, except for the presence or absence of an allele conferring resistance to the pesticide etoxazole (i.e., a single nucleotide mutation, I1017F, on the chitin synthase one gene, CHS1; Van Leeuwen et al. 2012), were used in the experiments. Etoxazole has no effect on the number of eggs produced but it disrupts the production of chitin, preventing egg hatching and halting juvenile development, with no effect on the adults (Van Leeuwen et al. 2012). This mutation is recessive and sufficient to reach high resistance levels, but, in a pesticide‐free environment, is linked with costs in developmental time (Bajda et al. 2018). The procedure used to create these populations is depicted in Figure 1 and fully described in the sections below.

#### 2.1.1 Creation of the *Wi.SS* Populations

Five *Wolbachia*‐infected homozygous etoxazole‐susceptible (‘*Wi.SS*’) population replicates were initially created in 2015, each by merging six compatible *Wolbachia*‐infected populations collected in the region of Lisbon, Portugal, in 2013 (Zélé, Santos, Olivieri, et al. 2018; Zélé, Santos, et al. 2020). Each population replicate was initially created with a census population size of 200 mated females and subsequently maintained with discrete generations at this same population size (*cf*. detailed procedure for the creation and maintenance of these populations, called ‘Infected controls; iC’, in Rodrigues, Zélé, et al. 2022). Full homozygosity for the etoxazole‐susceptible allele was subsequently confirmed by PCR‐RFLP (as described in Van Leeuwen et al. 2012) for each of the field‐derived populations and each of the *Wi.SS* replicates.

#### 2.1.2 Creation of the *Wi.RR* Populations

Five *Wolbachia*‐infected homozygous etoxazole‐resistant (‘*Wi.RR*’) population replicates were created in 2018 by introgression of an etoxazole‐resistant allele into each of the *Wi.SS* population replicates (Figure 1 and detailed procedure in Figure S1). The etoxazole‐resistant allele originated from the homozygous etoxazole‐resistant (RR) red lab strain *SB9*, collected in Crete, Greece, in 2006 (Van Leeuwen et al. 2012). As coinfection with *Wolbachia* and *Rickettsia* was detected in this strain (using the multiplex PCR procedure described in Zélé, Weill, and Magalhães 2018), and because these endosymbionts may cause reproductive incompatibilities with the *Wolbachia* strain that is present in the *Wi.SS* populations, *SB9* was treated with a rifampicin solution at 0.05%, w/v for one generation as described in Zélé, Altıntaş, et al. (2020) to create the symbiont‐free donor parent strain *SB9.rif*. We then used a backcross procedure ensuring the complete introgression of the etoxazole‐resistant allele from the *SB9.rif* donor strain into each of the *Wi.SS* recurrent parent populations, with an estimated retention of 99.6% of their nuclear background, and complete retention of their mitochondrial background and *Wolbachia* infection.

At the onset of the backcross procedure, *SB9.rif* males were crossed with females from each of the *Wi.SS* population replicates to obtain a 100% heterozygous F1 female progeny (hence all ‘*Wi.RS*’: *Wolbachia*‐infected with a resistant and a susceptible allele). Given haplodiploidy in spider mites and because the allele conferring resistance to etoxazole is recessive, these females were isolated as virgin to produce ‘*Wi.S*’ and ‘*Wi.R*’ F2 haploid males (note that this step replaces the usual self‐cross step for the introgression of recessive alleles in diploid organisms). Resistant F2 males (*Wi.R*) were then selected by being placed on etoxazole‐soaked cotton (diluted in water at 0.5 g/L) during the development of the male offspring and subsequently backcrossed with females from the recurrent parent population *Wi.SS* to produce a new generation of heterozygous females (i.e., the first generation of backcrossed females; BC_1_). Heterozygous BC_1_ females were, as their grandmothers, isolated as virgin to produce a new generation of males (i.e., the progeny of ‘noncrossed’ females, NC_1_), which were again selected for etoxazole resistance during their development. This procedure was repeated for six additional backcrosses (BC_2_NC_2_ to BC_7_NC_7_). At the end of the procedure, a subset of the progeny resulting from the last generation of backcross (*Wi.RS* females and *Wi.S* males; BC_7_) was self‐crossed (SC_1_) to obtain *Wi.RS* and *Wi.SS* females, which were crossed with NC_7_ selected cousins. Their offspring (SC_2_) was then exposed to etoxazole to select the homozygous resistant *Wi.RR* females and *Wi.R* males that founded the replicated populations ‘*Wi.RR*’. A census population size of at least 200 females and 100 males was used at each step of the procedure (*cf*. detailed procedure in Figure S1). The concentration of etoxazole used is higher than the LC100 seen in Van Leeuwen et al. (2012), having the same effect on our populations as it led them to be 100% homozygous resistant.

#### 2.1.3 Creation and Merge of the *Wu.SS* and *Wu.RR* Populations

The five *Wi.SS* and the five *Wi.RR* population replicates (each with a population size of 200 females) were subsequently treated for one generation with rifampicin (0.05% w/v), following the procedure described in Zélé, Altıntaş, et al. (2020). Each newly created *Wu.SS* and *Wu.RR* population replicates were subsequently maintained in discrete generations (population size of 200 females) in the absence of antibiotics (i.e., under the same conditions as the untreated populations). Three generations later, the presence/absence of *Wolbachia* and that of the etoxazole‐resistant allele were confirmed in a pool of 100 females from each *Wi.SS*, *Wi.RR*, *Wu.SS* and *Wu.RR* population replicate by multiplex PCR (see Zélé, Weill, and Magalhães 2018) and PCR‐RFLP (see Van Leeuwen et al. 2012), respectively. Forty‐seven generations after the creation of the replicate populations, and two generations before the onset of the experiments (i.e., in September 2020), all replicates from either the *Wu.SS* or the *Wu.RR* populations were merged to create the susceptible and resistant base populations used in this study, respectively.

### 2.2 Rearing Conditions

Since their creation and merging, both *Wu.SS* and *Wu.RR* were maintained in large numbers (> 2000) under continuous generations on entire plants. These populations were kept on bean leaves (*Phaseolus vulgaris*, variety Contender, provided by Germisem, Portugal) under controlled conditions (25°C; 70% humidity; photoperiod of 16L:8D). Bean plants were germinated and grown in an isolated and herbivore‐free room for 14 days under controlled conditions (photoperiod of 16L at 25°C:8D at 20°C) before being infested with spider mites.

### 2.3 Experimental Setup

#### 2.3.1 Effect of High Developmental Temperatures on Life‐History Traits

First, we characterised the impact of four developmental temperatures above the control temperature in the laboratory (control temperature: 25°C, high temperatures: 33°C, 35°C, 36°C and 37°C) on developmental time, survival and fertility of pesticide‐susceptible spider mites (from *Wu.SS*). These high temperatures are representative of the weather conditions that spider mites are exposed to in the field, particularly during summer months in Portugal, where the populations were collected. Between June and August, spider mites are found at high densities in the field (Zélé, Santos, Godinho, et al. 2018), being exposed to temperatures ranging, on average, from 16.5°C to 31.4°C (IPMA 2023a), with two heat waves occurring in August, each lasting 7 days and reaching 46.4°C (IPMA 2023b). The control temperature of 25°C is representative of the mean temperatures found during summer months in Portugal (IPMA 2023a) and it is the temperature used to maintain the populations in the laboratory since they were collected, so they are adapted to it.

To assess the effect of temperature on developmental time and juvenile survival, mite cohorts were created by placing on a bean leaf patch (ca. 75 cm^2^) 100 mated females for 2 days at 25°C, so they could oviposit synchronously. Once adult, the offspring of those females was transferred to one of the tested temperatures (25°C, 33°C, 35°C, 36°C or 37°C) for 3 days, after which a maximum of 32 offspring females were individually placed on bean leaf discs (2.55 cm^2^) to oviposit for 24 h at the same temperature. Egg development was followed daily, and the number of days elapsed until the first adult male and female offspring emerged (i.e., developmental time), as well as the number of eggs that reached adulthood (i.e., number of surviving juveniles) was recorded.

Due to logistic constraints, three independent assays were performed, always including the control temperature (25°C): (i) 25°C, 33°C and 37°C; (ii) 25°C and 35°C; and (iii) 25°C and 36°C. In all cases, replicates with damaged (i.e., harmed during manipulation) mothers were excluded from the analyses. Additionally, in the analysis of all independent variables, patches with females that did not lay eggs were excluded. Overall, we analysed 67, 65 and 82 replicates (i.e., patches) for female developmental time, male developmental time and number of surviving juveniles, respectively (see Table S1 for more details). Due to the low number of adult females surviving and ovipositing at 37°C, this temperature was not included in the statistical analyses of developmental time and number of surviving juveniles (see Table S2).

To determine the impact of heat on fertility, we focused only on female fertility, as we assumed that male fertility would also be hindered at the temperature females are affected, since males generally suffer from a reduction in fertility at lower temperatures than females in several arthropod species (David et al. 2005; Sales et al. 2018; Iossa 2019; Zwoinska et al. 2020; van Heerwaarden and Sgrò 2021). Cohorts were created with mated females as before, but they were directly maintained at one of the tested temperatures (25°C, 33°C, 35°C, 36°C or 37°C). Before the offspring reached adulthood, deutonymph quiescent males and females were isolated separately to ensure virginity. Once adults (ca. 24 h later), a maximum of 40 pairs of virgin females and males were isolated on bean leaf discs (2.55 cm^2^) to mate and oviposit. Five treatments were established: pairs where both individuals developed at 25°C (control; ♀25 × ♂25) and pairs where only the female developed at one of the high temperatures (♀33 × ♂25, ♀35 × ♂25, ♀36 × ♂25 and ♀37 × ♂25). Pairs were placed at the female developmental temperature and maintained during 24 h, after which males were discarded. Females were left to lay eggs for two more days, after which they were also discarded, and the eggs were counted. Leaf discs were placed at 25°C from this moment onwards. The survival status of both male and female was recorded until individual removal. Once reaching adulthood, offspring were counted and removed 12 and 14 days after the onset of oviposition. Daily fecundity and offspring number were used as proxies for female fertility.

As in the previous experiment, temperature treatments were distributed in three assays: (i) 25°C, 33°C and 37°C; (ii) 25°C and 35°C; and (iii) 25°C and 36°C. For the first assay, we performed two blocks, which occurred in two consecutive days. The other two assays were done in a single block. In all cases, replicates with individuals forming the mating pairs that were damaged during manipulation were excluded from the analyses. In addition, patches with females that did not lay eggs were excluded from the analysis of offspring number. Overall, we analysed 140 and 117 replicates (i.e., patches) for daily fecundity and offspring number, respectively (see Table S1 for more details).

#### 2.3.2 Interaction Between Pesticide Resistance and Response to High Developmental Temperature

To test if the effect of high developmental temperature on developmental time, survival and fertility differed depending on the resistance status of individuals, we exposed both resistant (*Wu.RR*) and susceptible (*Wu.SS*) populations to either 25°C or 36°C and repeated the experiments described above. In this case, when testing fertility, we measured both sexes, with offspring number being used to access female fertility and sex ratio to access male fertility, as only daughters contain the genetic material of the father. Here, females were given 4 days to oviposit instead of three, and four treatments were established to separately test the response of males and females to high developmental temperature: pairs where both individuals developed at 25°C (control; ♀25 × ♂25), and pairs where each or both sexes developed at 36°C (♀36 × ♂25, ♀25 × ♂36 and ♀36 × ♂36, respectively). Pairs were maintained at 25°C when both individuals developed at that temperature. Otherwise, they were kept at 36°C until the sex that developed at 36°C was removed, at which time they were moved to 25°C. When both sexes developed at 36°C, the transfer to 25°C occurred when the last individual of the pair was removed. All other experimental details are as above.

Replicates were excluded according to the same criteria as described above. Overall, we analysed the developmental time of females and males from 97 and 95 patches, respectively, the number of surviving juveniles from 114 patches and the number and sex of offspring from 233 patches (see Table S1 for more details).

### 2.4 Statistical Analyses

All statistical analyses were performed using the software R (version 4.0.3; R Core Team 2020). Depending on the data and error structure, we performed linear models (LM), linear mixed‐effects models (LMM), generalised linear models (GLM) and generalised mixed‐effects models (GLMM) implemented in *lme4* (version 1.1.26; Bates et al. 2015) and *glmmTMB* (version 1.0.2.1; Brooks et al. 2017; see Tables S3 and S4). Maximal models were simplified by removing nonsignificant terms (*p* > 0.05) from the highest‐ to the lowest‐order interaction (Crawley 2012). The significance of each explanatory variable was determined by the chi‐squared tests for discrete distributions, and Wald F tests for continuous distributions (Bolker et al. 2009). Graphic representations of the data were produced with the software package *ggplot2* (version 3.3.3; Wickham 2016).

#### 2.4.1 Effect of High Developmental Temperatures on Life‐History Traits

The effect of developmental high temperatures on juvenile traits was assessed by analysing two traits: the number of days from egg until adulthood (i.e., number of days the first male or female took to reach adulthood in a patch), used as a proxy for developmental time of each sex; and the number of surviving juveniles (i.e., number of juveniles surviving until the adult stage; Table S3). The effect of developmental heat stress on female fertility was determined by analysing daily fecundity and offspring number (Table S3).

As temperatures were not tested simultaneously, we analysed the differences of each high temperature to the control temperature. For that, within each assay, the mean trait value measured at the control temperature was subtracted from the individual trait values measured at the high temperature tested (trait value at the high temperature—$\bar{x}$ trait value at 25°C). When the assay was done in more than one block, the mean of the control temperature was calculated within each block, to account for that variability. The new variable was used as dependent variable in the subsequent models.

All variables were analysed using LM models with a Gaussian error distribution, except for the number of surviving juveniles that was analysed using a LMM model (Table S3). The data were box cox transformed (female developmental time: *λ* = 1.94; male developmental time: *λ* = 0.73; number of surviving juveniles: *λ* = 0.91; *MASS* package, *boxcox* function; version 7.3.53; Venables and Ripley 2002) to improve the fit of the model for each variable, except for daily fecundity and offspring number. To analyse the impact of temperature on developmental time and number of surviving juveniles, the developmental temperature (25°C, 33°C, 35°C or 36°C) was included in the models as fixed factor (Table S3). Fecundity was added as a random factor in the model testing the impact of temperature on the number of surviving juveniles, to account for the variation in the number of eggs laid (Table S3). In the analyses of daily fecundity and offspring number, the type of cross between males and females from different developmental temperatures (♀25 × ♂25, ♀33 × ♂25, ♀35 × ♂25, ♀36 × ♂25 or ♀37 × ♂25) was included in the model as fixed factor (Table S3).

To determine if the difference of each temperature to the control was significant, we used the function *summary* and took the value that reports the difference of the first temperature listed in the dataset to an intercept of zero (which in this case corresponds to the mean trait value at control temperature; Table 2a). This was repeated for all temperatures by reordering this variable in the dataset. To compare life‐history traits at the different high temperatures, a posteriori contrasts with Bonferroni corrections of significant explanatory variables were made using the *emmeans* package (version 1.5.3; Length 2020; Table 2b).

#### 2.4.2 Interaction Between Pesticide Resistance and Response to High Developmental Temperature

To test whether developmental heat stress differentially affected individuals with different resistance status in a sex‐specific manner, we analysed four traits: developmental time, the number of surviving juveniles, the offspring number and the offspring sex ratio (Table S4).

Developmental time and the number of surviving juveniles were analysed using GLM and GLMM models, respectively, with a Poisson error distribution (Table S4). The developmental temperature of the individuals (25°C or 36°C), their resistance status (susceptible or resistant) and the interaction between these two factors were included in all models as fixed factors. Fecundity was included as a random factor in the analysis of the number of surviving juveniles.

The offspring number was analysed using a GLMM model with a quasi‐Poisson error distribution (Table S4). Offspring sex ratio, analysed as the proportion of daughters, was computed using the function *cbind* with the number of daughters and sons as arguments (Table S4). This variable was analysed with a GLMM model with a beta‐binomial error distribution and a parameter to account for zero inflation (ziformula ~1; package *glmmTMB*). In the analysis of both offspring number and sex ratio, the developmental temperature of the female and of the male (25°C or 36°C, in both variables), the resistance status of the pair (susceptible or resistant) and all possible interactions were included as fixed factors. We paired males and females from two different cohorts (i.e., males and females could be 9 or 10 days old when developed at 25°C, or 7 or 8 days old when developed at 36°C), so the age of each sex was included as a random factor (Table S4). A posteriori contrasts with Bonferroni corrections were made using the *emmeans* package (Length 2020; Table 4).

## 3 Results

### 3.1 Effect of High Developmental Temperatures on Life‐History Traits

**FIGURE 2:**
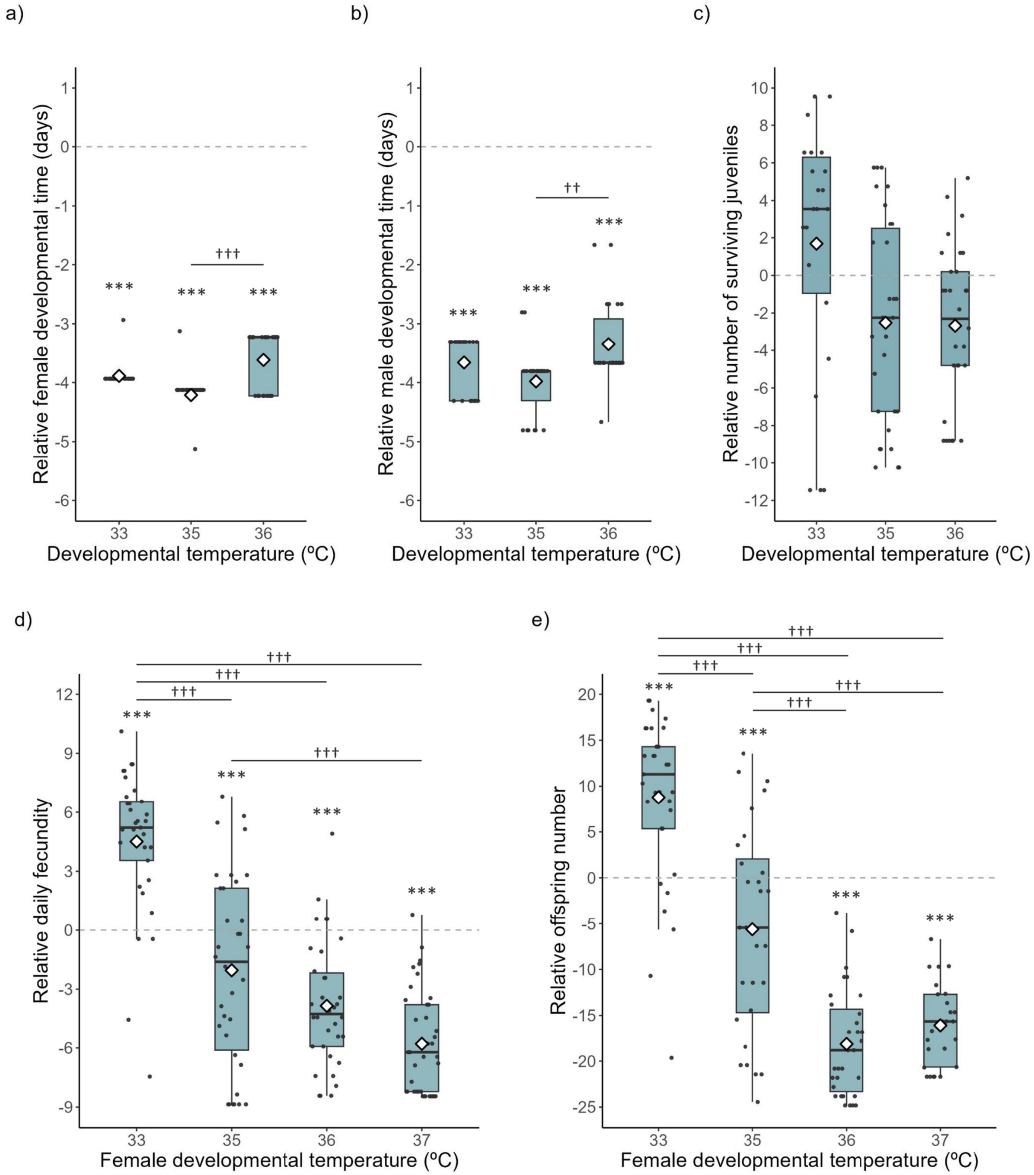
Effect of high developmental temperatures on life‐history traits. Difference between each tested temperature (33°C, 35°C, 36°C and 37°C) and the mean value at 25°C (control; dashed line). Developmental time, defined as the day the first female (a) and male (b) in a patch reached adulthood, and number of surviving juveniles, defined as the number of eggs that reached adulthood (c), were tested by allowing females that had developed at 25°C to oviposit at each indicated temperature. Data obtained at 37°C were excluded due to lack of replication. Female fertility was tested by placing females developed at each indicated temperature with males developed at 25°C, and allowing them to oviposit at their developmental temperature to assess their mean daily fecundity as the number of eggs laid per day (d), and offspring production as the number of eggs that reached adulthood (e). Boxplots display a median line, interquartile range (IQR) boxes, 1.5*IQR whiskers and data points. Black dots represent individual replicates; white diamonds represent the mean values per conditions tested. Asterisks indicate significant differences between tested temperatures and the control (25°C). Daggers indicate significant differences between tested temperatures. The number of symbols indicate the level of statistical significance: ^††^ *p* ≤ 0.01, ^***^ or ^†††^ *p* ≤ 0.001.

The developmental time of males and females was affected by the temperature they developed at (Table 1), with both sexes developing faster at all high temperatures than at the control temperature. Also, individuals developed significantly faster at 35°C than at 36°C (Table 2; Figure 2a,b). In contrast, the number of surviving juveniles, accounting for the total number of eggs produced, was not affected by the developmental temperature of individuals (Table 1; Figure 2c).

**TABLE 1:** Effect of high developmental temperatures on life‐history traits.

Var. of interest	Explanatory var.	df (df residuals)	*F*	*p*
Developmental time (♀)	**Developmental temperature**	**2 (10.651)**	**4.08**	**< 0.001**
Developmental time (♂)	**Developmental temperature**	**2 (6.153)**	**4.491**	**0.004**
Number of surviving juveniles	Developmental temperature	2 (31.880)	2.2084	0.126
Daily fecundity	**Temperature of pair**	**3 (51.478)**	**2074.07**	**< 0.001**
Offspring number	**Temperature of pair**	**3 (70.384)**	**14176.4**	**< 0.001**

*Note:* ‘Developmental time (♀)’ and ‘Developmental time (♂)’: Number of days the first female or male, respectively, took to reach adulthood in a patch; ‘Number of surviving juveniles’: Number of juveniles surviving until the adult stage; ‘Daily fecundity’: Mean number of eggs laid per day, representing female fertility; ‘Offspring number’: Number of eggs that reached adulthood laid by females exposed to different temperatures (proxy for female fertility). ‘Developmental temperature’: Temperature at which eggs were exposed until adulthood; ‘Temperature of pair’: Developmental temperature of both females and males paired to mate and oviposit (offspring developed at 25°C). Statistically significant terms (*p* ≤ 0.05) are represented in bold. For temperatures to be comparable, traits were analysed by their difference between each tested temperature (33°C, 35°C, 36°C and 37°C) and the mean value at 25°C (control).

Abbreviations: df, degrees of freedom; *F*, the sum of squares obtained from the *F*‐test.

**TABLE 2:** A posteriori contrasts of significant explanatory variables for the effect of high developmental temperatures on life‐history traits.

Var. of interest	Difference to the control (°C)	*T* ratio	*p*
(a)			
Developmental time (♀)	**33**	**−40.712**	**< 0.001**
	**35**	**−46.158**	**< 0.001**
	**36**	**−39.64**	**< 0.001**
Developmental time (♂)	**33**	**−27.097**	**< 0.001**
	**35**	**−31.599**	**< 0.001**
	**36**	**−25.998**	**< 0.001**
Daily fecundity	**♀33**	**7.067**	**< 0.001**
	**♀35**	**−3.250**	**0.001**
	**♀36**	**−6.114**	**< 0.001**
	**♀37**	**−9.871**	**< 0.001**
Offspring number	**♀33**	**6.156**	**< 0.001**
	**♀35**	**−3.631**	**< 0.001**
	**♀36**	**−12.327**	**< 0.001**
	**♀37**	**−9.810**	**< 0.001**

Var. of interest	Comparison (°C)	*T* ratio	*p*
(b)			
Developmental time (♀)	33–35	2.454	0.051*
	33–36	−2.050	0.133
	**35–36**	**−4.610**	**< 0.001**
Developmental time (♂)	33–35	1.733	0.264
	33–36	−1.671	0.299
	**35–36**	**−3.507**	**0.003**
Daily fecundity	**♀33–♀35**	**7.315**	**< 0.001**
	**♀33–♀36**	**9.325**	**< 0.001**
	**♀33–♀37**	**11.883**	**< 0.001**
	♀35–♀36	2.025	0.269
	**♀35–♀37**	**4.361**	**< 0.001**
	♀36–♀37	2.268	0.150
Offspring number	**♀33–♀35**	**6.841**	**< 0.001**
	**♀33–♀36**	**13.136**	**< 0.001**
	**♀33–♀37**	**11.441**	**< 0.001**
	**♀35–♀36**	**5.860**	**< 0.001**
	**♀35–♀37**	**4.637**	**< 0.001**
	♀36–♀37	−0.937	1.000

*Note:* For temperatures to be comparable, traits were analysed by their difference between each tested temperature (33°C, 35°C, 36°C and 37°C) and the mean value at 25°C (control). (a) To compare high temperatures to the control (25°C), the function *summary* was used to obtain the difference between each temperature and an intercept of zero, which corresponds to the mean trait value at the control temperature. (b) A posteriori contrasts with Bonferroni corrections were done to interpret the difference between high temperatures (33°C, 35°C, 36°C or 37°C). ‘*T* ratio’: The *t*‐test value obtained in each comparison. Comparisons were made between offspring developed at 25°C, 33°C, 35°C or 36°C (‘25°C’, ‘33°C’, ‘35°C’ and ‘36°C’), and between pairs with males developed at 25°C and females developed at 25°C, 33°C, 35°C, 36°C or 37°C (‘♀25°C’, ‘♀33°C’, ‘♀35°C’, ‘♀36°C’ and ‘♀37°C’). Statistically significant terms (*p* ≤ 0.05) are represented in bold.

* Marginally significant values.

Female fertility was affected by developmental temperature (Table 1), with both daily fecundity and the offspring number being higher at 33°C and lower at 35°C, 36°C and 37°C, compared with the control temperature (Table 2a; Figure 2d,e). In both cases, 36°C and 37°C showed the lowest values compared with the other temperatures (Table 2b; Figure 2d,e).

In sum, fertility was most affected at 36°C and 37°C. However, at 37°C adult survival was drastically hindered (ca. 10% survival after 3 days of exposure; Table S2). Therefore, we used 36°C as the sublethal temperature in subsequent tests.

### 3.2 Interaction Between Pesticide Resistance and Response to High Developmental Temperature

**FIGURE 3:**
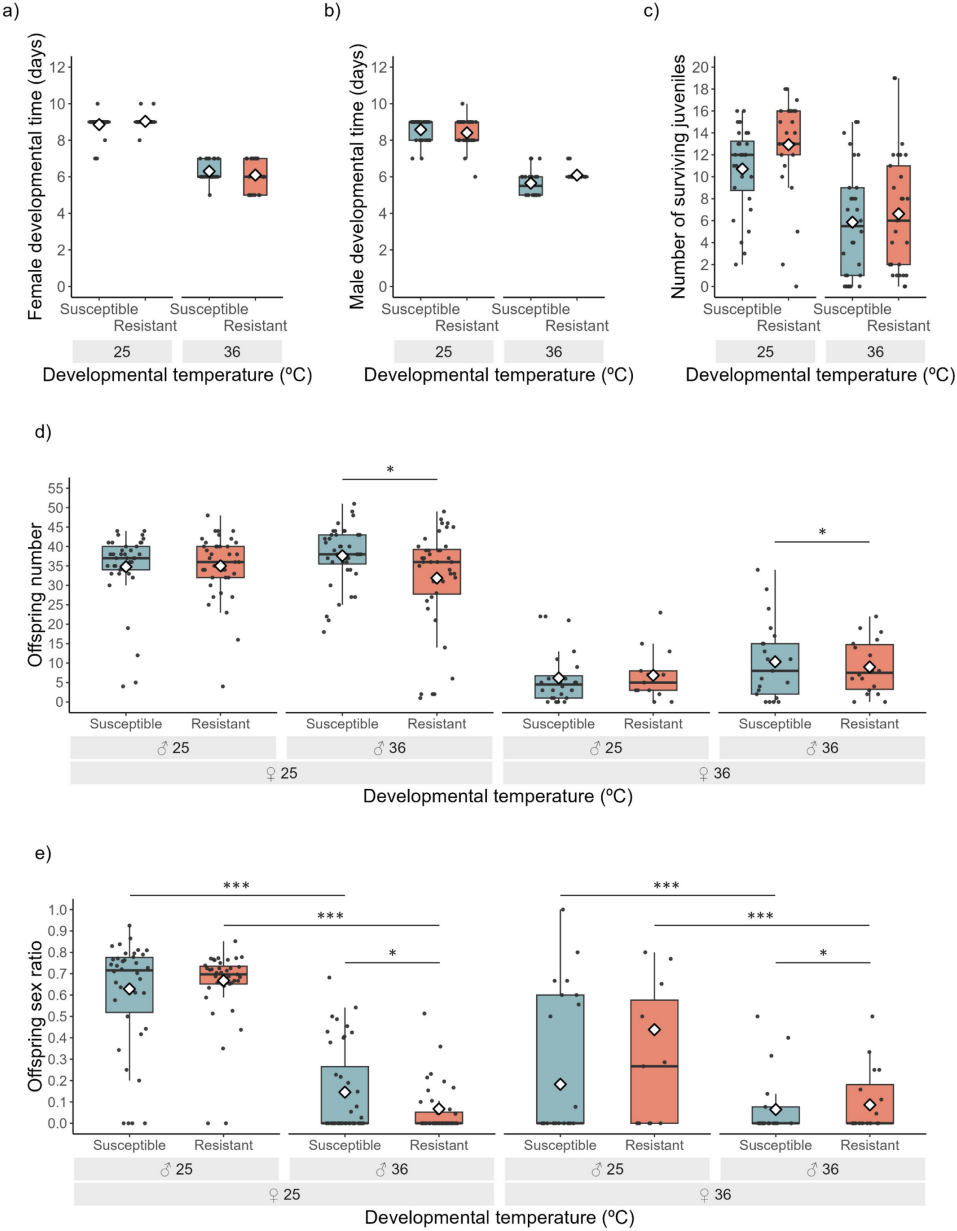
Sex‐specific effects of the interaction between pesticide resistance and high developmental temperature. Effect of 36°C during development on populations susceptible (blue bars) or resistant (red bars) to the pesticide etoxazole. Developmental time was measured by the day the first female (a) and male (b) in a patch reached adulthood, the number of surviving juveniles by the number of eggs that reached adulthood (c), female fertility by the offspring number (d), and male fertility by the offspring sex ratio (proportion of daughters) (e). Boxplots display a median line, interquartile range (IQR) boxes, 1.5*IQR whiskers and data points. Black dots represent individual replicates; white diamonds represent the mean values per conditions tested. Asterisks indicate significant differences between male developmental temperature and resistance status (*p ≤ 0.05, ***p ≤ 0.001).

The developmental time and number of surviving juveniles were not affected by the interaction between developmental temperature and resistance status (Table 3). Instead, these traits were equally affected by high developmental temperature (36°C) in individuals from susceptible and resistant populations (Table 3; Figure 3a–c).

**TABLE 3:** Effect of pesticide resistance on the response to high developmental temperature.

Var. of interest	Explanatory var.	df	*χ* ^2^	*p*
Developmental time (♀)	**Developmental temperature**	**1**	**23.578**	**< 0.001**
	Resistance status	1	0.001	0.977
	Developmental temperature*Resistance status	1	0.118	0.731
Developmental time (♂)	**Developmental temperature**	**1**	**22.175**	**< 0.001**
	Resistance status	1	0.032	0.857
	Developmental temperature*Resistance status	1	0.359	0.549
Number of surviving juveniles	**Developmental temperature**	**1**	**62.199**	**< 0.001**
	Resistance status	1	0.047	0.829
	Developmental temperature*Resistance status	1	0.072	0.788
Offspring number	**♀ Temperature**	**1**	**243.924**	**< 0.001**
	♂ Temperature	1	2.492	0.114
	Resistance status	1	0.291	0.590
	♂ Temperature*Resistance status	**1**	**5.227**	**0.022**
	♀ Temperature*Resistance status	1	1.471	0.225
	♀ Temperature*♂ temperature	1	3.170	0.075
	♀ Temperature*♂ temperature*Resistance status	1	0.207	0.649
Offspring sex ratio	**♀ Temperature**	**1**	**10.957**	**< 0.001**
	**♂ Temperature**	**1**	**66.215**	**< 0.001**
	Resistance status	1	0.181	0.670
	♂ Temperature*Resistance status	**1**	**4.895**	**0.027**
	♀ Temperature*Resistance status	1	1.661	0.197
	♀ Temperature*♂ temperature	1	0.322	0.571
	♀ Temperature*♂ temperature*Resistance status	1	3.062	0.080

*Note:* ‘Developmental time (♀)’ and ‘Developmental time (♂)’: Number of days the first female or male, respectively, took to reach adulthood in a patch; ‘Number of surviving juveniles’: Number of juveniles surviving until the adult stage; ‘Offspring number’: Number of eggs that reached adulthood coming from crosses with females and males developed at high or control temperatures (proxy for female fertility); ‘Offspring sex ratio’: Proportion of adult daughters in the offspring (proxy for male fertility). ‘Developmental temperature’: Temperature at which eggs were exposed until adulthood; ‘♀ temperature’: Developmental temperature of the females paired; ‘♂ temperature’: Developmental temperature of the males paired; ‘Resistance status’: Resistance or susceptibility to etoxazole; ‘Fecundity’: Number of eggs laid for 4 days. Statistically significant terms (*p* ≤ 0.05) are represented in bold.

Abbreviations: df, degrees of freedom; *χ* ^2^, chi‐square value obtained in each analysis.

Fertility was affected by the interaction between the developmental temperature of the male and the resistance status of the pair (Table 3). Indeed, resistant males produced fewer offspring and a lower proportion of daughters than susceptible ones, but only when they developed at 36°C (Table 4; Figure 3d,e). In turn, the female developmental temperature affected offspring number and sex ratio similarly in the two populations, as all pairs with females developed at 36°C produced fewer offspring and a lower proportion of daughters than all pairs with females developed at 25°C (Table 3; Figure 3d,e).

**TABLE 4:** A posteriori contrasts of significant explanatory variables for the interaction between pesticide resistance and response to high developmental temperature.

Var. of interest	Comparison	*T* ratio	*p*
Offspring number	Susceptible ♂25°C–Susceptible ♂36°C	−1.659	0.394
	Resistant ♂25°C–Resistant ♂36°C	1.579	0.463
	Resistant ♂25°C–Susceptible ♂25°C	0.540	1.000
	**Resistant ♂36°C–Susceptible ♂36°C**	**−2.705**	**0.029**
Offspring sex ratio	**Susceptible ♂25°C–Susceptible ♂36°C**	**8.560**	**< 0.001**
	**Resistant ♂25°C–Resistant ♂36°C**	**8.137**	**< 0.001**
	Resistant ♂25°C–Susceptible ♂25°C	−0.426	1.000
	**Resistant ♂36°C–Susceptible ♂36°C**	**−2.641**	**0.035**

*Note:* A posteriori contrasts with Bonferroni corrections were done to interpret the significant effect of the fixed factors. ‘*T* ratio’: The *t*‐test value obtained in each comparison. Comparisons were made between males developed at 25°C or 36°C (‘♂25’ and ‘♂36’) and that were susceptible or resistant to etoxazole (‘Susceptible’ and ‘Resistant’). Statistically significant terms (*p* ≤ 0.05) are represented in bold.

## 4 Discussion

Our results show that all but one life‐history traits measured in individuals from the susceptible population (i.e., number of surviving juveniles) responded to high developmental temperatures in a nonlinear manner (Figure 2). At 36°C, fertility was affected the most, while survival was as yet not compromised (Figure 2; Table 1). We thus selected this temperature to test for sex‐specific effects of temperature on individuals with different resistant status. Developmental time and number of surviving juveniles were affected by temperature but not by resistance status (Figure 3; Table 3). In contrast, reproductive traits of resistant and susceptible individuals were affected by high developmental temperatures in a sex‐specific manner (Figure 3; Table 3). Indeed, for males that developed at high temperature, matings involving resistant males resulted in fewer offspring and more sons than matings involving susceptible ones. Still, offspring number and sex ratio did not differ between susceptible and resistant females developed at that same temperature (Table 4).

We found that the response of several traits to temperature was nonlinear, with daily fecundity and offspring number peaking at 33°C and the developmental time of both sexes reaching its lowest at 35°C (Table 2). Evidence of nonlinear responses in this and other arthropods towards increasing temperatures (Umoetok Akpassam, Iloba, and Udo 2017; Zou et al. 2018; Sales et al. 2018; Zwoinska et al. 2020) and other environmental variables (Godinho et al. 2018; Godinho, Branquinho, and Magalhães 2023; Guedes, Rix, and Cutler 2022) are common. In *Tetranychus*, some studies show a decrease in developmental time with temperature (Kasap 2004; Praslička and Huszár 2004; Bayu et al. 2017; Zou et al. 2018), while other studies report a decrease followed by an increase in developmental time with rising temperatures (Riahi et al. 2013; Hasanvand, Jafari, and Khanjani 2020; Farazmand 2020). A peak in daily fecundity has also been observed in previous work, although the temperatures for such peak differed between studies (Kasap 2004; Riahi et al. 2013; Zou et al. 2018). These differences may be related to the origin of the populations tested, given that populations from different climates are adapted to specific sets of temperatures (Deutsch et al. 2008; García‐Robledo et al. 2016). Furthermore, it is important to note that population growth in polyphagous species is highly dependent on the host plant species (Praslička and Huszár 2004; Clissold and Simpson 2015). Therefore, our results may be specific to the plant used here. Clearly, more studies are needed to understand the origin and consequences of nonlinear responses to temperature.

Several studies show that resistance to pesticide can trade‐off with life‐history traits in pesticide‐free environments (e.g., Carrière et al. 1994; Okuma et al. 2018; Yan et al. 2014), including in *T. urticae* (Stumpf and Nauen 2002; Sato et al. 2005; Nicastro, Sato, and Da Silva 2010, 2011) and specifically when resistant to etoxazole (Stocco, Sato, and Santos 2016; Bajda et al. 2018). However, in our study we found no cost of etoxazole resistance at control temperature for any of the traits measured (Table 3), which should contribute to the maintenance of pesticide resistance in these populations long after pesticide use. One possible explanation for the discrepancy between studies could be the existence of different genetic bases contributing to resistance to etoxazole (e.g., Van Leeuwen et al. 2012; Ilias, Vontas, and Tsagkarakou 2014; Adesanya et al. 2018), some entailing costs and others not. Another possibility could be that the observed costs are associated with, but not due to, resistance. First, there could be non‐genetic differences between resistant and susceptible populations that were responsible for the negative association between resistance and life‐history traits. For instance, pesticide‐resistant mosquitoes (*Culex pipiens*) are often more heavily infected with *Wolbachia* than susceptible ones, with the costs associated with the resistance phenotype being due in part to the infection and not to resistance (Duron et al. 2006). In turn, the differences between resistant and susceptible populations could be genetic, the costs being due to differences in genetic background. This was observed in *Culex quinquefasciatus*, in which the fitness costs associated with resistance to organophosphate identified when comparing strains from different locations were strongly reduced after backcrossing the resistant gene into the genetic background of the susceptible strain (Amin and White 1984). Here, we eliminated the possibility of nongenetic differences due to symbionts by treating the populations with antibiotics. Unlike some previous studies (e.g., Okuma et al. 2018; Stocco, Sato, and Santos 2016; Yan et al. 2014), we also minimised genetic differences among resistant and susceptible populations by using an introgression procedure. Still, because we are studying genetically variable populations, the resistant population may have evolved after introgression to compensate for an existing cost of resistance. Such phenomenon was observed, for instance, in the sheep blowfly *Lucilia cuprina*, which evolved a modifier gene that eliminated the cost of resistance to the insecticide diazinon after long‐term exposure (circa 120 generations) (McKenzie, Whitten, and Adena 1982; McKenzie and Clarke 1988). Such hypothesis would explain the discrepancy between our study and that of Bajda et al. (2018), who found a cost of the same resistance gene in *T. urticae* after performing a similar introgression procedure but on isogenic lines, thereby preventing evolution. All in all, this suggests that the genetic background in which resistance to etoxazole is inserted plays a role in the expression of costs, but more studies should be performed to confirm this possibility.

While resistance was not costly at control temperature, with the individuals from resistant and susceptible populations showing similar values for all life‐history traits, fertility was differently affected at 36°C depending on resistance status of the individuals. Indeed, the reduction in the number of offspring and in the proportion of daughters felt after exposure to the developmental high temperature was steeper in pairs involving resistant individuals than in those involving susceptible individuals (Table 4). These results are in accordance with a few other studies done on other crop pests such as *Plutella xylostella* and *Nilaparvata lugens* (Li et al. 2007; Zhang et al. 2015; Yang et al. 2018), showing that pesticide resistance trades‐off with heat tolerance. Such a cost of pesticide resistance on fertility at high temperatures should reduce the selective advantage of resistant individuals, which in turn could slow the evolution of resistance to insecticides, thus increasing the efficacy of crop management by pesticide application. However, to the best of our knowledge, none of these previous studies has investigated possible sex‐specific responses, thus risking overestimating the cost of resistance.

Here, we find that the trade‐off between pesticide resistance and heat tolerance occurs in males but not in females. Several studies have shown that male thermal fertility limits are the best predictors of extinction in several species (Sales et al. 2018; Iossa 2019; Parratt et al. 2021; van Heerwaarden and Sgrò 2021). This suggests that the fertility toll suffered by resistant spider mite males can foster the exclusion of resistance from the population. On the contrary, the observed sex‐specific response to temperature may buffer the costs of pesticide resistance, assuming resistant females have the chance to mate with migrant males from cooler locations or mate multiply to compensate for sterility. This last strategy has been observed in several arthropod species, including spider mites (Berger et al. 2011; Sutter et al. 2019; Vasudeva et al. 2021; Baur et al. 2022; Costa, Magalhães, and Rodrigues 2023).

Given sex‐specific responses, the sex‐determination system of the species will also influence the outcome of resistance in the population. First, because of haplodiploidy in spider mites, high temperature affected not only the number but also the sex ratio of the offspring. Each of these traits entails different consequences for population dynamics. For instance, while both fecundity and sex ratio influence population growth rate, they may do so in different ways, with the effects of sex ratio depending on population structure but those of fecundity not being influenced by this factor (Metz and Gyllenberg 2001). Second, resistance to etoxazole in spider mites is recessive (Van Leeuwen et al. 2012). Therefore, haploid males will express the resistance cost as soon as they carry one resistance allele, promoting a quick elimination of resistance (Carrière 2003), which would not be the case in diploid organisms, including diploid females from haplodiploid species.

Overall, we show that, in a pesticide‐free environment, climate change can delay the growth of spider mite populations feeding on bean plants as temperatures rise, negatively affecting several life‐history traits, with pesticide‐resistant males being more affected by temperature than susceptible males. While this is good news from a pest management standpoint, the complex expectations stemming from its sex‐determination system and the possible buffering effect of females coupled with the decrease in developmental time of both sexes at high temperature may counter such forces. Indeed, decreased developmental time due to heat should result in an increased number of generations per crop period (Manikandan, Kennedy, and Geethalakshmi 2013; Bayu et al. 2017; Zou et al. 2018). Thus, in environments with pesticides, populations in warmer temperatures should evolve pesticide resistance at a faster rate than those at colder temperatures (Bebber, Ramotowski, and Gurr 2013; Skendžić et al. 2021). In turn, for populations in pesticide‐free environments, temperature‐induced shorter generations could lead to a faster evolution of the compensation of a resistance cost. Alternatively, individuals with susceptible alleles may replace those with costly resistant ones at a faster rate, leading to a swift reduction in resistance in natural populations, as seen in *Halotydeus destructor* (Umina et al. 2024). Importantly, an increase in the number of generations per crop period should also accelerate adaptation to high temperature itself (Foucault et al. 2018; González‐Tokman et al. 2020).

Whether such adaptation will erode the differences between resistant and susceptible males, as seen in the control temperature, is yet to be tested. Ultimately, effort should be put into studying the evolution of populations exposed to both stressors, temperature and pesticide to better understand and effectively predict the dynamics of crop pests in a changing world.

## Conflicts of Interest

The authors declare no conflicts of interest.

## Supporting information


Appendix S1.

## Data Availability

The data that support the findings of this study are openly available in Figshare at https://doi.org/10.6084/m9.figshare.22596832.v1 (Costa et al. 2024).
