# Supplementary material for: A Sex‐Specific Trade‐Off Between Pesticide Resistance and Tolerance to Heat‐Induced Sterility in Tetranychus urticae
Source: Evol Appl. 2024 Sep 26;17(9):e70014. doi: 10.1111/eva.70014 (PMC11424881; doi:10.1111/eva.70014)
Supplement: Supplementary file 1 — Appendix S1. [file EVA-17-e70014-s001.docx]

**Supplementary materials**

**A sex-specific trade-off between pesticide resistance and tolerance to heat-induced sterility in *Tetranychus urticae***

**Table of contents**

**Table S1** Number of replicates analysed in each experiment performed …………………………….. 2

**Table S2** Survival and oviposition of adult females exposed to different temperatures .……………... 4

**Table S3** Description of the statistical models used to analyse the effect of high developmental temperatures on life-history traits ..……………………………………………………………………. 5

**Table S4** Description of the statistical models used to analyse the interaction between pesticide resistance and the response to high developmental temperature ...……………………………………. 6

**Fig. S1** Detailed backcross procedure used to create the *Wi.RR* population replicates ………………. 7

**Table S1** **Number of replicates analysed in each experiment performed.** a) the effect of high developmental temperatures on life-history traits; b) the effect of the interaction between pesticide resistance and high developmental temperature on life-history traits. “Resistance status”: the individuals tested could be from a population resistant or susceptible to the pesticide etoxazole; “Treatment”: developmental temperatures (25, 33, 35, 36 or 37°C in a), and 25 or 36°C in b)) of the offspring (in variables “Developmental time (♀) and (♂)” and “Number of surviving juveniles”) or of the female and male crossed (in variables “Daily fecundity”, “Offspring number” and “Offspring sex ratio”). In all response variables, replicates with damaged (*i.e.,* harmed during manipulation) mothers (in variables “Developmental time (♀) and (♂)” and “Number of surviving juveniles”) or damaged individuals forming the mating pairs (in variables “Daily fecundity”, “Offspring number” and “Offspring sex ratio”) were excluded from the analyses; in all response variables except “Daily fecundity”, females that did not lay eggs were excluded from the analyses. ^a^ Due to the low number of replicates (see Table S2), 37°C was not considered in the statistical analyses. ^b^ Due to logistic constraints, temperatures were not tested simultaneously; instead, three assays were performed, always including the control temperature, and testing 33 and 37 together, 35 alone or 36°C alone; as such, three values are provided, each corresponding to a different assay following the previously stated order. ^c^ The values of offspring number and sex ratio were taken from the same patches

**a)**

| **Response Variable** | **Resistance status** | **Treatment** | **No. of replicates** |
| --- | --- | --- | --- |
| Developmental time (♀) ^a^ | Susceptible | 25 | 31/32/31 ^b^ |
|  |  | 33 | 21 |
|  |  | 35 | 23 |
|  |  | 36 | 23 |
| Developmental time (♂) ^a^ | Susceptible | 25 | 29/31/30 ^b^ |
|  |  | 33 | 20 |
|  |  | 35 | 23 |
|  |  | 36 | 22 |
| Number of surviving juveniles ^a^ | Susceptible | 25 | 31/32/31 ^b^ |
|  |  | 33 | 22 |
|  |  | 35 | 30 |
|  |  | 36 | 30 |
| Daily fecundity | Susceptible | ♀25 x ♂25 | 37/36/36 ^b^ |
|  |  | ♀33 x ♂25 | 33 |
|  |  | ♀35 x ♂25 | 34 |
|  |  | ♀36 x ♂25 | 34 |
|  |  | ♀37 x ♂25 | 39 |
| Offspring number | Susceptible | ♀25 x ♂25 | 37/36/33 ^b^ |
|  |  | ♀33 x ♂25 | 33 |
|  |  | ♀35 x ♂25 | 28 |
|  |  | ♀36 x ♂25 | 31 |
|  |  | ♀37 x ♂25 | 25 |

**b)**

| **Response Variable** | **Resistance status** | **Treatment** | **No. of replicates** |
| --- | --- | --- | --- |
| Developmental time (♀) | Susceptible | 25 | 28 |
|  |  | 36 | 20 |
|  | Resistant | 25 | 28 |
|  |  | 36 | 21 |
| Developmental time (♂) | Susceptible | 25 | 26 |
|  |  | 36 | 20 |
|  | Resistant | 25 | 27 |
|  |  | 36 | 22 |
| Number of surviving juveniles | Susceptible | 25 | 28 |
|  |  | 36 | 28 |
|  | Resistant | 25 | 29 |
|  |  | 36 | 29 |
| Offspring number and  Offspring sex ratio ^c^ | Susceptible | ♀25 x ♂25 | 38 |
|  |  | ♀25 x ♂36 | 40 |
|  |  | ♀36 x ♂25 | 24 |
|  |  | ♀36 x ♂36 | 21 |
|  | Resistant | ♀25 x ♂25 | 39 |
|  |  | ♀25 x ♂36 | 40 |
|  |  | ♀36 x ♂25 | 13 |
|  |  | ♀36 x ♂36 | 18 |

**Table S2** **Survival and oviposition of adult females exposed to different temperatures.** Females developed at 25°C in cohorts were maintained at 25°C or were transferred to one of the four high temperatures (33, 35, 36 or 37°C) for 3 days, after which they were isolated to oviposit for 24 hours at the same temperature. A minimum of 60 and a maximum of 200 females were transferred to high temperature for 3 days. “No. alive ♀ at the time of isolation”: a maximum of 32 alive adult females (*i.e.*, maximum number of replicates created) were isolated on a patch after 3 days at high temperature. “No. ovipositing ♀ (% ovipositing ♀)”: number and percentage of females that laid at least one egg during the oviposition period. At 37°C, the survival and percentage of ovipositing of adult females was very low, which led to the exclusion of the data obtained at this temperature from the experiment. ^a^ Due to logistic constraints, temperatures were not tested simultaneously; instead, three assays were performed, always including the control temperature. 33 and 37°C were tested together, and 35 and 36°C were tested alone. As such, three values are provided, each corresponding to a different assay following the previously stated order.

| Temperature | No. alive ♀ at the time of isolation | No. ovipositing ♀  (% ovipositing ♀) |
| --- | --- | --- |
| 25°C | 32/32/32 ^a^ | 31/32/31 ^a^ (96.9/100/96.9%) |
| 33°C | 26 | 22 (84.6%) |
| 35°C | 32 | 30 (93.8%) |
| 36°C | 32 | 32 (100%) |
| 37°C | 6 | 3 (50%) |

**Table S3** **Description of the statistical models used to analyse the effect of high developmental temperatures on life-history traits.** “Sample size”: total number of replicates included in each analysis. “Maximal model”: complete set of explanatory variables included in the model. “Minimal model”: model containing only the variables that were statistically significant; round brackets indicate that the variable was included as a random factor. Within R subroutine, “lm”: linear model, “lmer”: linear mixed-effects model. Square brackets indicate the error structure (“g”: gaussian). “Developmental temperature”: temperature at which eggs were exposed until adulthood; “Fecundity”: the difference between the number of eggs laid at a high temperature and the mean number of eggs laid at control temperature (25°C); “Temperature of pair”: developmental temperature of both females and males paired to mate and oviposit (offspring developed at 25°C). “$\bar{x}$”: mean trait value. ^a^ only includes replicates in which fecundity was above zero. ^b^ due to the low number of replicates (see Table S2), 37°C was not considered in the statistical analyses. Replicates with damaged (*i.e.,* harmed during manipulation) mothers or individuals forming the mating pairs were excluded in all analyses

| **Var. of interest** | **Response variable** | **Sample size** | **Maximal model** | **Minimal model** | **R subroutine [err struct.]** |
| --- | --- | --- | --- | --- | --- |
| Developmental time (♀) | Day of 1^st^ adult ♀ at high developmental temperature - $\bar{x}$(day of 1^st^ adult ♀ at 25°C) | 161 ^a, b^ | Developmental temperature | Developmental temperature | lm [g] |
| Developmental time (♂) | Day of 1^st^ adult ♂ at high developmental temperature - $\bar{x}$(day of 1^st^ adult ♂ at 25°C) | 155 ^a, b^ | Developmental temperature | Developmental temperature | lm [g] |
| Number of surviving juveniles | Adult offspring at high developmental temperature - $\bar{x}$(adult offspring at 25°C) | 176 ^a, b^ | Developmental temperature + (Fecundity) | Developmental temperature + (Fecundity) | lmer [g] |
| Daily fecundity | Daily fecundity from ♀ developed at high temperature - $\bar{x}$(daily fecundity from ♀ developed at 25°C) | 249 | Temperature of pair | Temperature of pair | lm [g] |
| Offspring number | Adult offspring from ♀ developed at high temperature - $\bar{x}$(adult offspring from ♀ developed at 25°C) | 223 ^a^ | Temperature of pair | Temperature of pair | lm [g] |

**Table S4** **Description of the statistical models used to analyse the interaction between pesticide resistance and the response to high developmental temperature.** “Sample size”: total number of replicates included in each analysis. “Maximal model”: complete set of explanatory variables included in the model. “Minimal model”: model containing only the variables that were statistically significant. Round brackets indicate that the variable was included as a random factor. Within R subroutine, “glm”: general linear model, “glmer” and “glmmTMB”: general mixed-effects models. Square brackets indicate the error structure used (“p”: Poisson; “qp”: quasi-Poisson; “bbI”: beta-binomial, accounting for zero inflation). “Developmental temperature”: temperature at which eggs were exposed until adulthood; “Resistance status”: resistance or susceptibility to etoxazole; “Fecundity”: number of eggs laid for four days; “♂”: number of sons; “♀”: number of daughters. “♀ temperature”: developmental temperature of the females paired with males to mate and oviposit (offspring developed at 25°C); “♂ temperature”: developmental temperature of the males paired with females to mate and oviposit (offspring developed at 25°C); “Age of male”: Age at which each male was paired; “Age of female”: Age at which each female was paired. All analyses include only replicates in which fecundity was above zero. Replicates with damaged (*i.e.,* harmed during manipulation) mothers or individuals forming the mating pairs were excluded all analyses

| **Var. of interest** | **Response variable** | **Sample size** | **Maximal model** | **Minimal model** | **R subroutine [err struct.]** |
| --- | --- | --- | --- | --- | --- |
| Developmental time (♀) | Day of first adult female | 97 | Developmental temperature *  Resistance status | Developmental temperature | glm [p] |
| Developmental time (♂) | Day of first adult male | 95 | Developmental temperature *  Resistance status | Developmental temperature | glm [p] |
| Number of surviving juveniles | Number of adult offspring | 114 | Developmental temperature *  Resistance status + (Fecundity) | Developmental temperature + (Fecundity) | glmer [p] |
| Offspring number | Number of adult offspring | 233 | ♀ temperature * ♂ temperature * Resistance status + (Age of male) + (Age of female) | ♀ temperature + ♂ temperature * Resistance status | glmmTMB [qp] |
| Offspring sex ratio | cbind (♀, ♂) | 233 | ♀ temperature * ♂ temperature * Resistance status + (Age of male) + (Age of female) | ♀ temperature + ♂ temperature * Resistance status | glmmTMB [bbI] |

**Fig. S1** **Detailed backcross procedure used to create the *Wi.RR* population replicates.** For each population replicate, F0 crosses (left panel) were performed by mixing 300 quiescent females (*i.e.,* female nymphs undergoing their last moulting stage) from each *Wi.SS* population box with 100 males from the SB9.rif donor strain, into a petri dish containing a bean leaf placed on water-saturated cotton. Females emerged as adult virgins and could mate for 3 days, after which 200 of them were transferred to an experimental box (14 × 14 × 20 cm) containing two 17-day-old bean plants whose stem was imbibed in wet cotton. Eleven days later, 300 quiescent females were collected from each experimental box for a first **‘no-cross’ (NC)** step. These females were placed without males (to remain virgin when emerging as adult) into a petri dish containing a bean leaf placed on water-saturated cotton and could lay eggs for 7 days (*T. urticae* is haplodiploid so virgin females produce male offspring). **Selection** was then applied to the offspring (all males) by transferring the bean leaf into a new petri dish containing cotton soaked with a 0.5g/L lethal concentration of etoxazole (trade name Borneo) (*cf.* van Leeuwen et al. 2012). Seven days later, 100 adult males (all resistant) were collected from this petri dish and **back-crossed (BC)** with 300 quiescent females collected from each *Wi.SS* population box (following the same procedure than for F0 crosses). The sequence ‘**no-cross, selection, backcross’** was then repeated for six additional generations. At the seventh generation of backcross (right panel), all remaining BC_7_ females (not isolated as virgin to produce NC_7_ males) were kept in the experimental box for 3 more days to emerge as adult and mate with BC_7_ males, *i.e.*, **‘self-cross’ (SC)**. Then, 400 of these females were transferred into two new experimental boxes (200 females per box, each with two fresh bean plants). Eleven days later, 600 quiescent SC_1_ females were collected from these two boxes and equally distributed across 4 petri dishes with 400 selected NC_7_ males. These females emerged as adult, mated and laid SC_2_ eggs for 7 days. The bean leaves carrying this SC_2_ progeny were then transferred into new petri dishes with an etoxazole solution (as described above) to select resistant individuals (*Wi.RR* females and *Wi.R* males). Ten days later, 200 adult mated females were collected from these petri dishes and transferred into a new experimental box without pesticide to found a *Wi.RR* replicate. This entire procedure was repeated independently for the 5 *Wi.SS* population replicate to create the 5 *Wi.RR* population replicates
